# Psychological Distress, Anxiety, Family Violence, Suicidality, and Wellbeing in Pakistan During the COVID-19 Lockdown: A Cross-Sectional Study

**DOI:** 10.3389/fpsyg.2022.830935

**Published:** 2022-03-15

**Authors:** Farah Yasmin, Hafsa Nazir Jatoi, Muhammad Saif Abbasi, Muhammad Sohaib Asghar, Sarush Ahmed Siddiqui, Hamza Nauman, Abdullah Khan Khattak, Muhammad Tanveer Alam

**Affiliations:** ^1^Department of Internal Medicine, Dow Medical College, Dow University of Health Sciences, Karachi, Pakistan; ^2^Department of Internal Medicine, Dow University of Health Sciences, Karachi, Pakistan; ^3^Department of Internal Medicine, Aga Khan University Hospital, Karachi, Pakistan; ^4^Department of Internal Medicine, Civil Hospital, Dow University of Health Sciences, Karachi, Pakistan

**Keywords:** COVID-19, psychological distress, anxiety, well-being, SARS-CoV-2

## Abstract

**Background and Objectives:**

The purpose of this study was to draw the attention toward the implications of COVID-19 and the related restrictions imposed worldwide especially in Pakistan. The primary objective was to highlight the levels of psychological distress, anxiety, family violence, suicidality, and well-being due to COVID-19 and the secondary objective was to associate it to social demographic factors.

**Materials and Methods:**

It is designed as a cross-sectional study by employing an online questionnaire in the English language and obtaining responses using a snowball sampling technique. We used three validated measures including Kessler Psychological Distress Scale (K10), Generalized Anxiety Disorder (GAD-7) index and World Health Organization Well-Being Index (WHO-5).

**Results:**

A sample of 420 participants was recruited from across Pakistan, with most participants were females (79%), students (89.8%) and belonging to Punjab (54%). Nearly one-fourth of the participants (23.8%) scored above the minimum value set for moderate or high psychological distress (K10 > 12). There was a higher prevalence of distress among females and resident of province Punjab. The majority of individuals reported that they were living with their family (94.5%) and more than half (52.6%) were neutral regarding their satisfaction with their living conditions. 40.5% believed that the lockdown has had a negative impact on their mental health. 31.4% have reported that they themselves have experienced abuse from a family member. 48.6% scored high on the GAD-7 scale and low wellbeing score was found among 80.2%. Students were found to be more vulnerable to mental illness and anxiety.

**Conclusion:**

With the lockdown restrictions, psychosocial distress has become prevalent in Pakistan.

## Introduction

Severe Acute Respiratory Syndrome-Coronavirus 2 (SARS-CoV-2) first emerged in Wuhan China, in December 2019 and has since affected 222 countries, with a total of 209,670,370 confirmed cases and 4,399,468 deaths globally as of 18th August 2021 (Worldometer Coronavirus, 2022). The increasing numbers suggest high transmissibility of the virus. Based on the reproductive numbers of novel coronavirus-19 (COVID-19), its estimated transmissibility is 4.1 (Wang et al., 2020). This suggests that with each confirmed case of COVID-19, there will be 4 new confirmed cases and inevitably, on March 11, 2020, the World Health Organization (WHO) declared the COVID-19 outbreak a global pandemic (Wang et al., 2020; World Health Organization [WHO], 2022). Following the declaration, many countries such as New Zealand initiated steps to control the spread of the virus such as implementing a nationwide lockdown. In Pakistan, the first case was confirmed on February 26, 2020, and a nation-wide lockdown was imposed on April 1, 2020 (Jawed, 2020). The lockdown helped curtail the spread of the virus and currently Pakistan, despite being densely populated, ranks 33rd in the list of COVID-19 affected countries (Worldometer Coronavirus, 2022). As of 26th November, the total number of confirmed cases reported in Pakistan are 1,283, 886 while the total number of deaths equals 28,704 (Worldometer Coronavirus, 2022).

Although strict nationwide lockdown was strategically effective in limiting the spread of the virus, it had negative implications on the mental well-being of individuals. Many studies have been conducted globally which highlight the impact of COVID-19 pandemic on the mental health and well-being of people, with most of these studies implying a surge in anxiety and depression in individuals due to the pandemic restrictions (Banna et al., 2020; Every-Palmer et al., 2020; Fornili et al., 2021). 30.3% of the 2,010 individuals surveyed in a cross-sectional study conducted in New Zealand reported moderate to severe psychological distress (Every-Palmer et al., 2020). Of these people, the majority had either lost their jobs, had lesser workload due to the pandemic, had previously reported mental illnesses or were at an increased risk of COVID-19 (Every-Palmer et al., 2020). Results of a study conducted in Italy showed an aggravation of all six domains under investigation: anxiety, depression, positive wellbeing, self-control, general health, and vitality. The difference in the index before and after the quarantine due to the pandemic was found out to be 15.1% (Fornili et al., 2021). A study from Bangladesh included 1,447 participants, of whom 59.7% reported to be experiencing stress symptoms, and 33.7% of the individuals reported anxiety symptoms of which 11.6% had severe anxiety symptoms. 59.7% faced depressive symptoms of which 13.2% were having severe symptoms (Banna et al., 2020). Evidently, the pandemic has also significantly affected the mental well-being of health-care workers. An assessment of anxiety and trust levels among Iranian health care workers revealed that 30.4% of health care workers had mild to moderate levels of anxiety while 21.3% had severe levels of anxiety. Regarding trust levels, lowest levels were found for social media users while highest levels were observed for TV viewers (Hasannia et al., 2021). Additionally, based on the results of another cross-sectional study assessing post-traumatic stress disorder (PTSD) symptoms, insomnia, and psychological distress amongst 500 Taiwanese health care workers, 15.4% had PTSD symptoms, 44.6% faced insomnia, 30.6% had high levels of anxiety, and 23.4% high stress levels (Lu et al., 2021).

Students were also severely affected by the pandemic. Studies conducted amongst students of Taiwan, Indonesia and Thailand revealed increased anxiety levels, the highest levels being observed in Thai students who were also found to have lesser trust in the available COVID-19 protocols to combat the disease. Significantly, amongst Taiwan students, international students were found to have higher anxiety levels compared to local students (Ahorsu et al., 2020, 2021).

The incidence of family violence also increased during the pandemic due to strict lockdowns, increased anxiety, financial instability and decline in provision of support services (Herbert et al., 2021). The United Nations Population Fund (UNFPA) and collaborators have suggested that in a 6-month lockdown due to the pandemic, there would be 31 million added cases of intimate partner violence and further 13 million child marriages by the year of 2030 [United Nations Population Fund, Avenir Health Johns Hopkins University; Victoria University (Australia), 2020]. These results point out the need for increased accessibility of support service providers for the families in lockdown due to COVID-19. Suicidality, which is closely associated with psychological distress, anxiety, family violence and well-being, has also increased significantly during the COVID-19 restrictions. Extrapolating data from previous viral outbreak of severe acute respiratory syndrome (SARS) in 2003, suicide rates are known to increase due to isolation and quarantine (Chan et al., 2006). According to previously conducted studies, the factors contributing toward this increase include the concern of getting the disease, passing the disease to others, mental instability, economic recession, and the absence of food and alcohol (Dsouza et al., 2020; Mamun and Griffiths, 2020; Mamun and Ullah, 2020). The fear of COVID-10 most likely stems from uncertainty of future career, reduced job security and satisfaction (Rajabimajd et al., 2021). Hence, it is not surprising that the suicide rates have increased worldwide during the COVID-19 pandemic. Based on a study conducted in Bangladesh, from 13,654 respondents, 8% reported suicide ideation during the pandemic (Islam et al., 2021).

In Pakistan, although similar events as mentioned above take place, most go unreported since there is a severe lack of awareness. This lack of awareness is basically due to a smaller number of studies conducted in Pakistan which could highlight the impact of COVID-19 on mental health and suicide rates. Extracting data from the limited literature available from Pakistan, a study designed to assess the impact of COVID-19 on mental health showed that increased psychological distress and anxiety are due to the fear of contracting the disease and due to the financial, social, and religious implications of the pandemic (Ali N. A. et al., 2021). The symptoms of anxiety and depression are also increased in people, especially health care workers. In Pakistan, a cross sectional survey results reported that in 1,094 participants, the median depression score was 5.00 and a median anxiety score was 8.00 suggesting that with the progressing state of the pandemic, there was a rise in mild levels of depression and anxiety among health care workers (Hayat et al., 2021). Furthermore, 90% of the women in Pakistan have reported having faced domestic violence (Baig et al., 2020). In March 2020, during the lockdown, 399 women were killed in Khyber Pakhtunkhwa while the police helpline only received 25 calls for help (Baig et al., 2020). Consequently, with a higher number of people facing mental health issues, financial uncertainty or domestic violence, suicide rates are also high in Pakistan. From January 2020 to May 2020, the number of reported suicide cases in Pakistan was 29, of which 16 were due to COVID-19 related issues (Mamun and Ullah, 2020). This represents the fact that Pakistan has a higher number of suicide cases reported than most other countries due to the stigma associated with suicide and mental health as this results in people not reaching out to service providers for help. This is also because there is a lack of knowledge about telemedicine amongst the population.

Our study draws attention to the implications of COVID-19 and the related restrictions imposed worldwide especially in Pakistan. The primary objective of the study was to highlight the levels of psychological distress, anxiety, family violence, suicidality, and well-being due to COVID-19 in Pakistan. The secondary objective of the study is to associate it to social demographic factors.

## Materials and Methods

### Participants and Recruitment Methods

Our study involved a considerable sample population of Pakistan aged between 18 and 70 years. We designed a cross-sectional study, and the results were obtained by employing an online questionnaire in the English language from 12th March 2021 to 30th July 2021, since it was unfeasible to conduct a nationwide survey on ground during the lockdown. The questionnaires were filled anonymously using a snowball sampling technique where each respondent was encouraged to share the survey with others. The primary focus was to include the general population of Pakistan for which the survey was shared to people of different provinces using the messenger application, WhatsApp, and social groups on Facebook. The total sample size was 420. Confidentiality of all respondents was maintained; they were informed of the purpose of the study in the participant information section of the questionnaire and were requested to provide informed consent on the first page of the survey. They had the freedom to not participate in the study and had the right to deny the use of their data for the research. The exclusion criteria were the completeness of the questionnaires; all incomplete questionnaires were disregarded.

### Measures and Outcomes

The questionnaire employed was structured and was divided into two sections: (1) socio demographic characteristics and (2) Self-perceived psychological state during the pandemic with regards to stress, anxiety, and depression. The participants were requested to answer the questions based on their experience in the last 4 weeks. The first section of the questionnaire included data about gender, age, education, current occupation, marital status, range of household income, province, presence of comorbidities, self-reported health status, and self-reported COVID-19 risk. We also evaluated self-reported body mass index (BMI), in the questionnaire since psychological well-being is related to self-perceived outlook (Schmidt and Martin, 2019). The participants were also requested to answer questions regarding their living conditions such as, who they lived with, satisfaction with their living circumstances, level of contact with people outside their homes and ease of communication and understanding amongst people of the household.

We used the Kessler Psychological Distress Scale (K10) to assess the psychological distress amongst our sample population. K10 scale is a 10-item questionnaire used to evaluate distress based on symptoms of anxiety and depression (Andrews and Slade, 2001). The respondents reported the frequency of their symptoms such as nervousness, fatigue, sadness, and hopelessness, on a 5-point Likert scale which ranged from 1 (none of the time) to 5 (all the time). At the end of their responses, the numbers are all added up for the total score on the K10 scale. The reported scores are in the range of 0–40. The cut off value of total scores was 12; scores above 12 (K10 > 12) hinted at the potential of moderate or high psychological distress. We employed the use of the K10 scale because it is a reliable and valid measure with a Cronbach’s α of more than 0.88 as seen in previous studies (Brooks et al., 2006; Fassaert et al., 2009; Bu et al., 2017).

The degree of anxiety symptoms was assessed using the Generalized Anxiety (GAD-7) index as it is a well validated and reliable measure, previously used in large sample populations (Spitzer et al., 2006; Every-Palmer et al., 2020). This scale has an exceptional consistency with a Cronbach’s α value of 0.92 (Spitzer et al., 2006). The participants were required to answer 7 questions about anxiety symptoms based on a 4-point Likert scale which ranged from 1 (not at all) to 4 (nearly every day). The range of scores for the GAD-7 index were from 0 to 21 and the cut off value was taken as 10; scores higher than 10 indicated severe anxiety.

To get an insight into the subjective well-being of respondents we used the 5-item World Health Organization Well-Being Index (WHO-5), which is widely used within public health and mental health research (Topp et al., 2015; Every-Palmer et al., 2020). The WHO-5 consists of 5 simple statements and the participant is asked how well each of the statements applies to him or her in the past 14 days (Topp et al., 2015). The respondents choose a number ranging from 0 (none of the time) to 5 (all the time). The total well-being score ranges from 0 to 25, with 25 indicating maximum well-being (Topp et al., 2015). For this study, we used 13 as the cut-off value; respondents who scored less than 13 on the WHO-5 scale were regarded as individuals with low mental well-being.

We asked respondents questions regarding family violence, suicidal ideation, and experience of silver linings during the pandemic which were adopted from methods of previous studies conducted. The questions pertained to self-experienced violence, witnessed violence, suicidal thoughts, suicidal plans, suicidal efforts, and any hopeful prospects experienced by the respondents (Ghazizadeh, 2005; Every-Palmer et al., 2020; Mamun, 2021). The only answer choices provided for the question regarding silver linings during the pandemic were “yes, for me,” “yes, for the society” or a “no.”

The survey was initially tested amongst the public online on a small-scale. Responses from 30 participants were gathered. Based on their feedback, we assessed our questionnaire in order to evaluate the general understanding of the questions by the respondents. This evaluation was considered when making further improvements to the questionnaire. The improved sections of the questionnaire included the use of improved scales of answers (addition of “somewhat better or better and easy/somewhat easy” in certain questions) as well as clear and concise instructions. We then utilized the revised survey for the purpose of our study.

### Ethical Approval

The study received ethical approval from the Institution’s Ethics Committee of the Dow University Ojha Hospital. Each participant had the right to withdraw from the study at any time. The possible risks and the purpose of the survey were thoroughly explained. Participants had to provide consent before filling out the questionnaire.

### Data Analysis

Microsoft Excel 2016 was used for data collection and assembled into Statistical Package for Social Sciences (SPSS) version 25.0 for data analysis. Categorical variables were assessed using frequencies and percentages, and their respective 95% confidence intervals through univariate analysis. Analytical statistics were performed with odds ratios (OR) and 95% confidence intervals, which were obtained using logistic regression.

## Results

### Characteristics of Study Participants

A sample of 420 participants was recruited from across Pakistan, with the largest sample being from Punjab (*n* = 230), followed by Sindh (*n* = 137). Demographic characteristics of the population are shown in Table 1. Most of the participants (99%) belonged to the 18–29 years age group and a higher number of respondents were females (79%). Prior to lockdown, 70.2% of the participants considered themselves to be of normal weight, while after the lockdown, only 61% of the respondents reported themselves to be of normal weight. The difference is not that significant so one could infer that no such change in self-reported weight gain could have affected the psychological responses. Supplementary Table 1 summarizes the psychosocial responses of the study participants.

**TABLE 1:** Demographic characteristics of the sample population (*n* = 420).

Variable	Frequency (n)	Percentages (%)	95% confidence interval
**Gender**			
Male	88	21.0	17.2–25.2%
Female	332	79.0	74.8–82.8%
**Age**			
18–29 years	416	99.0	97.6–99.7%
30–59 years	3	0.7	0.1–2.1%
> 60 years	1	0.2	0.0–1.3%
**Self-reported BMI (before pandemic)**			
Underweight	62	14.8	11.5–18.5%
Normal	295	70.2	65.6–74.6%
Overweight	58	13.8	10.7–17.5%
Obese	5	1.2	0.4–2.8%
**Self-reported BMI (after pandemic)**			
Underweight	76	18.1	14.5–22.1%
Normal	256	61.0	56.1–65.6%
Overweight	77	18.3	14.7–22.4%
Obese	11	2.6	1.3–4.6%
**Education**			
No formal education	1	0.2	0.0–1.3%
Primary/Secondary education	18	4.3	2.6–6.7%
Higher secondary/College	221	52.6	47.7–57.5%
University graduate	170	40.5	35.7–45.3%
Postgraduate or higher	10	2.4	1.1–4.3%
**Occupation**			
Unemployed	8	1.9	0.8–3.7%
Government/Private employee	13	3.1	1.7–5.2%
Student	377	89.8	86.5–92.5%
Healthcare worker	22	5.2	3.3–7.8%
**Marital status**			
Married	7	1.7	0.7–3.4%
Single	413	98.3	96.6–99.3%
**Household income**			
< 20,000 Rupee	33	7.9	5.5–10.9%
21,000–50,000 Rupee	67	16.0	12.6–19.8%
51,000–100,000 Rupee	119	28.3	24.1–32.9%
101,000–200,000 Rupee	114	27.1	22.9–31.7%
> 200,000 Rupee	87	20.7	16.9–24.9%
**Region**			
Sindh	137	32.6	28.2–37.3%
Punjab	230	54.8	49.9–59.6%
KPK	47	11.2	8.3–14.6%
Balochistan	6	1.4	0.5–3.1%
**Comorbidities**			
Present	65	15.5	12.2–19.3%
Absent	355	84.5	80.7–87.8%
**Self-reported health status**			
Excellent/Very good	78	18.6	15.0–22.6%
Good/Average	245	58.3	53.5–63.1%
Fair/Poor	97	23.1	19.1–27.4%
**Self-reported COVID-19 risk**			
I have already had the coronavirus	73	17.4	13.9–21.4%
I don’t think I will get the coronavirus	172	41.0	36.2–45.8%
I think I will get a mild case of the coronavirus	158	37.6	33.0–42.4%
I think I will get a severe case of the coronavirus	17	4.0	2.4–6.4%

*KPK, Khyber Pakhtunkhwa; n, number of subjects.*

### Psychological Distress

Almost one-fourth of the participants (23.8%) scored above the minimum value set for moderate or high psychological distress (K10 > 12) (Table 2). Prevalence among the females (*p* = 0.05), and presence of comorbidities (*p* = 0.021) were found significant. Correlation is also observed between deteriorating self-reported health and prevalence of psychological distress. There was a higher prevalence of distress among the participants from Punjab.

**TABLE 2:** Logistic regression analysis of moderate/high psychosocial distress scores on K10 index.

Variable	K10 score > 12 (moderate or high)			Odds ratio (OR)	95% Confidence interval (95% CI)	*p*-value
	
	Frequency (n)	Percentages (%)	95% confidence interval (95% CI)			
**Gender**						
Male	60	18.8	14.6–23.5%	1.000	−	−
Female	260	81.3	76.5–85.4%	1.685	1.003–2.832	0.049
**Education**						
No formal education*	1	0.3	0.0–1.7%	−	−	−
Primary/Secondary education	10	3.1	1.5–5.7%	1.000	−	−
Higher secondary/College	178	55.6	50.0–61.2%	3.312	1.234–8.891	0.017
University graduate	125	39.1	33.7–44.6%	2.222	0.826–5.982	0.114
Postgraduate or higher	6	1.9	0.7–4.0%	1.200	0.250–5.768	0.820
**Occupation**						
Unemployed	6	1.9	0.7–4.0%	1.000	−	−
Government/Private employee	9	2.8	1.3–5.3%	0.750	0.103–5.470	0.777
Student	291	90.9	87.2–93.8%	1.128	0.224–5.690	0.884
Healthcare worker	14	4.4	2.4–7.2%	0.583	0.094–3.603	0.562
**Marital status**						
Married	3	0.9	0.2–2.7%	0.227	0.050–1.033	0.055
Single	317	99.1	97.3–99.8%	1.000	−	−
**Household income**						
< 20,000 Rupee	23	7.2	4.6–10.6%	0.876	0.364–2.110	0.768
21,000–50,000 Rupee	44	13.8	10.2–18.0%	0.729	0.366–1.452	0.368
51,000–100,000 Rupee	102	31.9	26.8–37.3%	2.286	1.139–4.585	0.020
101,000–200,000 Rupee	88	27.5	22.7–32.7%	1.289	0.678–2.451	0.438
> 200,000 Rupee	63	19.7	15.5–24.5%	1.000	−	−
**Region**						
Sindh	97	30.3	25.3–35.7%	1.000	−	−
Punjab	184	57.5	51.9–63.0%	1.649	1.011–2.692	0.045
KPK	35	10.9	7.7–14.9%	1.203	0.567–2.551	0.630
Balochistan	4	1.3	0.3–3.2%	0.825	0.145–4.684	0.828
**Comorbidities**						
Present	57	17.8	13.8–22.5%	2.492	1.146–5.422	0.021
Absent	263	82.2	77.5–86.2%	1.000	−	−
**Self-reported health status**						
Excellent/Very good	49	15.3	11.5–19.7%	1.000	−	−
Good/Average	185	57.8	52.2–63.3%	1.825	1.059–3.143	0.030
Fair/Poor	86	26.9	22.1–32.1%	4.627	2.126–10.070	< 0.001
**Self-reported COVID-19 risk**						
I have already had the coronavirus	53	16.6	12.7–21.1%	0.166	0.021–1.289	0.086
I don’t think I will get the coronavirus	125	39.1	33.7–44.6%	0.166	0.021–1.332	0.091
I think I will get a mild case of the coronavirus	126	39.4	34.0–45.0%	0.246	0.031–1.925	0.182
I think I will get a severe case of the coronavirus	16	5.0	2.9–8.0%	1.000	−	−
**Living circumstances**						
With others	314	98.1	96.0–99.3%	1.000	−	
Alone	6	1.9	0.7–4.0%	1.068	0.212–5.377	0.936
**Satisfaction with living circumstances**						
Dissatisfied/Neutral	250	78.1	73.2–82.5%	1.587	0.863–2.919	0.138
Satisfied/Very satisfied	70	21.9	17.5–26.8%	1.000	−	
**Lost job during the lockdown**						
Yes	12	3.7	2.0–6.5%	1.438	0.716–2.886	0.307
No/Never had a job	308	96.3	93.5–98.0%	1.000	−	
**Do you have a medical condition that makes you more vulnerable to COVID-19 infection?**						
No/Prefer not to say	217	67.8	62.4–72.9%	1.000	−	
Yes	103	32.2	27.1–37.6%	1.899	1.103–3.269	0.021
**History of any mental illness diagnosed by a doctor or psychologist?**						
No/Prefer not to say	272	85.0	80.6–88.7%	1.000	−	
Yes	48	15.0	11.3–19.4%	2.029	0.926–4.449	0.077
During the lockdown, have you seriously thought about ending your own life?						
No/Prefer not to say	234	73.1	67.9–77.9%	1.000	−	
Yes	86	26.9	22.1–32.1%	8.821	3.148–24.714	< 0.001
**During the lockdown, have you made plans/attempts to end your own life?**						
No/Prefer not to say	262	81.9	77.2–85.9%	1.000	−	
Yes	58	18.1	14.1–22.8%	5.313	1.878–15.029	0.002
**During lockdown, have you experienced any abuse as a result of an action from a family member?**						
No/Prefer not to say	195	60.9	55.4–66.3%	1.000	−	
Yes	125	39.1	33.7–44.6%	8.516	3.825–18.960	< 0.001
**During lockdown, have you been a witness to any abuse in your “bubble”?**						
No/Prefer not to say	237	74.1	68.9–78.8%	1.000	−	
Yes	83	25.9	21.2–31.1%	17.160	4.139–71.145	< 0.001

*Could not compute because of low number of responses. n, number of subjects; K10, Kessler Psychological Distress Scale.

### Living Circumstances and Interaction With Family

The majority of individuals reported that they were living with their family (94.5%) and more than half (52.6%) were neutral regarding their satisfaction with their living conditions. Regardless of this positive attitude toward their living situation, apart from 37 people (8.8%), the feeling of loneliness and isolation was felt by most of the respondents at some point during the lockdown.

### Mental Health and Suicidal Thoughts

Among the participants, 40.5% believed that the lockdown has had a negative impact on their mental health while 32.6% reported that there had not been a significant difference. During the lockdown 21.4% (95% CI: 17.6, 25.7) of the participants had seriously thought about ending their life. Data supports a correlation between worsening mental health and suicidal ideation.

### Family Harm

Totally 132 of the 420 participants have reported that they themselves have experienced abuse from a family member (31.4%) and the family harm has mostly been in the form of physical assault (34.8%), sexual assault (4.5%) and insults, harassment, or threatening behavior (33.3%). Worsening mental health and higher anxiety disorder scores were observed in individuals who faced abuse during lockdown.

### Silver Linings

Most of the respondents thought that lockdown has had positive impacts either for them personally (42.1%) or for the society as a whole (18.3%).

### Anxiety and Wellbeing

Close to half of the respondents (48.6%) scored higher than 10 on the GAD-7 scale, which was the cut-off value for the anxiety score to be regarded as high, whereas a low wellbeing score was found among 80.2% (95% CI 76.1–83.9%) of the individuals. Anxiety and mental wellbeing results are shown in Tables 3, 4 respectively. Students were more likely to score less on the WHO-5 index (*p* = 0.035). Individuals with history of mental illness scored significantly high on the GAD-7 index.

**TABLE 3:** Logistic regression analysis of anxiety disorder on GAD-7 index.

Variable	GAD > 10 (high anxiety score)			Odds ratio (OR)	95% Confidence interval (95% CI)	*p*-value
	
	Frequency (n)	Percentages (%)	95% confidence interval (95% CI)			
**Gender**						
Male	37	17.1	12.4–22.8%	1.000	−	−
Female	179	82.9	77.2–87.6%	1.613	1.003–2.593	0.049
**Education**						
No formal education*	0	0.0	−	−	−	−
Primary/Secondary education	6	2.8	1.0–5.9%	1.000	−	−
Higher secondary/College	120	55.6	48.7–62.3%	2.376	0.861–6.558	0.095
University graduate	85	39.4	32.8–46.2%	2.000	0.718–5.575	0.185
Postgraduate or higher	5	2.3	0.8–5.3%	2.000	0.412–9.712	0.390
**Occupation**						
Unemployed	2	0.9	0.1–3.3%	0.208	0.030–1.467	0.115
Government/Private employee	8	3.7	1.6–7.2%	1.000	−	−
Student	194	89.8	85.0–93.5%	0.663	0.213–2.062	0.687
Healthcare worker	12	5.6	2.9–9.5%	0.750	0.185–3.034	0.750
**Marital status**						
Married	1	0.5	0.0–2.6%	0.153	0.018–1.286	0.153
Single	215	99.5	97.4–100.0%	1.000	−	−
**Household income**						
< 20,000 Rupee	18	8.3	5.0–12.9%	1.120	0.501–2.502	0.782
21,000–50,000 Rupee	24	11.1	7.3–16.1%	0.521	0.271–1.001	0.050
51,000–100,000 Rupee	69	31.9	25.8–38.6%	1.288	0.739–2.246	0.372
101,000–200,000 Rupee	60	27.8	21.9–34.3%	1.037	0.593–1.813	0.898
> 200,000 Rupee	45	20.8	15.6–26.9%	1.000	−	−
**Region**						
Sindh	63	29.2	23.2–35.7%	1.000	−	−
Punjab	131	60.6	53.8–67.2%	1.554	1.016–2.378	0.042
KPK	20	9.3	5.7–13.9%	0.870	0.446–1.698	0.870
Balochistan	2	0.9	0.1–3.3%	0.587	0.104–3.314	0.587
**Comorbidities**						
Present	44	20.4	15.2–26.4%	2.229	1.273–3.903	0.005
Absent	172	79.6	73.6–84.8%	1.000	−	−
**Self-reported health status**						
Excellent/Very good	22	10.0	6.5–15.0%	1.000	−	−
Good/Average Fair/Poor	125 69	57.9 31.9	51.0–64.5% 25.8–38.6%	2.652 4.627	1.525–4.610 3.241–12.141	0.001 < 0.001
**Self-reported COVID-19 risk**						
I have already had the coronavirus	40	18.5	13.6–24.4%	1.461	0.843–2.532	0.177
I don’t think I will get the coronavirus	78	36.1	29.7–42.9%		−	−
I think I will get a mild case of the coronavirus	88	40.7	34.1–47.6%	1.000	0.981–2.339	0.061
I think I will get a severe case of the coronavirus	10	4.6	2.2–8.3%	1.515 1.722	0.626–4.734	0.292
**Living circumstances**						
With others	212	98.1	95.3–99.5%	1.000	−	
Alone	4	1.9	0.5–4.7%	0.943	0.233–3.823	0.935
**Satisfaction with living circumstances**						
Dissatisfied/Neutral	172	79.6	73.6–84.8%	1.017	0.632–1.638	0.945
Satisfied/Very satisfied	44	20.4	15.2–26.4%	1.000	−	
**Lost job during the lockdown**						
Yes	8	3.7	1.6–7.2%	1.923	0.570–6.487	
No/Never had a job	208	96.3	92.8–98.4%	1.000	−	0.292
**Do you have a medical condition that makes you more vulnerable to COVID-19 infection?**						
No/Prefer not to say	148	68.5	61.9–74.7%	1.000	−	
Yes	68	31.5	25.3–38.1%	1.245	0.816–1.898	0.309
**History of any mental illness diagnosed by a doctor or psychologist?**						
No/Prefer not to say	179	82.9	77.2–87.6%	1.000	−	
Yes	37	17.1	12.4–22.8%	2.013	1.116–3.631	0.020
**During the lockdown, have you seriously thought about ending your own life?**						
No/Prefer not to say	144	66.7	60.0–72.9%	1.000	−	
Yes	72	33.3	27.1–40.0%	5.167	2.950–9.049	< 0.001
**During the lockdown, have you made plans/attempts to end your own life?**						
No/Prefer not to say	167	77.3	71.1–82.7%	1.000	−	
Yes	49	22.7	17.3–28.9%	4.311	2.260–8.223	< 0.001
**During lockdown, have you experienced any abuse as a result of an action from a family member?**						
No/Prefer not to say	127	58.8	51.9–65.4%	1.000	−	
Yes	89	41.2	34.6–48.1%	2.624	1.703–4.042	< 0.001
**During lockdown, have you been a witness to any abuse in your “bubble”?**						
No/Prefer not to say	153	70.8	64.3–76.8%	1.000	−	
Yes	63	29.2	23.2–35.7%	3.406	2.003–5.792	< 0.001

*Could not compute because of low number of responses. n, number of subjects; GAD-7, Generalized Anxiety Disorder Assessment.

**TABLE 4:** Logistic regression analysis of low mental well-being scores on WHO-5 index.

Variable	WHO-5 score < 13 (low mental well-being)			Odds ratio (OR)	95% Confidence interval (95% CI)	*p*-value
	
	Frequency (n)	Percentages (%)	95% confidence interval (95% CI)			
**Gender**						
Male	68	20.2	16.0–24.9%	1.000	−	−
Female	269	79.8	75.1–84.0%	1.256	0.771–2.219	0.443
**Education**						
No formal education*	1	0.3	0.0–1.6%	−	−	−
Primary/Secondary education	16	4.7	2.7–7.6%	1.000	−	−
Higher secondary/College	190	56.4	50.9–61.7%	0.766	0.168–3.497	0.731
University graduate	126	37.4	32.2–42.8%	0.358	0.079–1.620	0.182
Post graduate or higher	4	1.2	0.3–3.0%	0.083	0.012–0.580	0.012
**Occupation**						
Unemployed	4	1.2	0.3–3.0%	1.000	−	−
Government/Private employee	8	2.4	1.0–4.6%	2.667	0.500–14.217	0.251
Student Healthcare worker	309 16	91.7 4.7	88.2–94.4% 2.7–7.6%	4.544 1.600	1.109–18.623 0.270–9.490	0.035 0.605
**Marital status**						
Married	2	0.6	0.1–2.1%	0.093	0.018–0.489	0.005
Single	335	99.4	97.9–99.9%	1.000	−	−
**Household income**						
< 20,000 Rupee	24	7.1	4.6–10.4%	1.000	−	−
21,000–50,000 Rupee	47	13.9	10.4–18.1%	0.881	0.348–2.228	0.789
51,000–100,000 Rupee	99	29.4	24.6–34.6%	1.856	0.751–4.585	0.180
101,000–200,000 Rupee	95	28.2	23.4–33.3%	1.875	0.754–4.662	0.176
> 200,000 Rupee	72	21.4	17.1–26.1%	1.800	0.698–4.639	0.224
**Region**						
Sindh	110	32.6	27.7–37.9%	1.000	−	−
Punjab	188	55.8	50.3–61.2%	1.099	0.642–1.881	0.732
KPK	36	10.7	7.6–14.5%	0.803	0.363–1.780	0.590
Balochistan	3	0.9	0.2–2.6%	0.245	0.047–1.284	0.096
**Comorbidities**						
Present	56	16.6	12.8–21.0%	1.639	0.775–3.465	0.196
Absent	281	83.4	79.0–87.2%	1.000	−	−
**Self-reported health status**						
Excellent/Very good	54	16.0	12.3–20.4%	1.000	−	−
Good/Average	196	58.2	52.7–63.5%	1.778	1.002–3.155	0.049
Fair/Poor	87	25.8	21.2–30.8%	3.867	1.717–8.710	0.001
**Self-reported COVID-19 risk**						
I have already had the coronavirus	60	17.8	13.9–22.3%	1.000	−	−
I don’t think I will get the coronavirus	133	39.5	34.2–44.9%	0.739	0.368–1.485	0.395
I think I will get a mild case of the coronavirus	129	38.3	33.1–43.7%	0.964	0.468–1.985	0.920
I think I will get a severe case of the coronavirus	15	4.5	2.5–7.2%	1.625	0.331–7.989	0.550
**Living circumstances**						
With others	330	97.9	95.8–99.2%	1.000	−	
Alone	7	2.1	0.8–4.2%	1.739	0.211–14.335	0.607
**Satisfaction with living circumstances**						
Dissatisfied/Neutral	266	78.9	74.2–83.2%	1.316	0.700–2.473	0.395
Satisfied/Very satisfied	71	21.1	16.8–25.8%	1.000	−	
**Lost job during the lockdown**						
Yes	12	3.6	1.9–6.1%	1.449	0.695–3.020	0.322
No/Never had a job	325	96.4	93.9–98.1%	1.000	−	
**Do you have a medical condition that makes you more vulnerable to COVID-19 infection?**						
No/Prefer not to say	237	70.3	65.1–75.2%	1.000	−	
Yes	100	29.7	24.8–34.9%	1.101	0.645–1.878	0.725
**History of any mental illness diagnosed by a doctor or psychologist?**						
No/Prefer not to say	293	86.9	82.9–90.4%	1.000	−	
Yes	44	13.1	9.6–17.1%	0.889	0.446–1.770	0.737
**During the lockdown, have you seriously thought about ending your own life?**						
No/Prefer not to say	259	76.9	72.0–81.3%	1.000	−	
Yes	78	23.1	18.7–28.0%	1.782	0.919–3.455	0.087
**During the lockdown, have you made plans/attempts to end your own life?**						
No/Prefer not to say	284	84.3	79.9–88.0%	1.000	−	
Yes	53	15.7	12.0–20.1%	4.356	0.571–33.213	0.156
**During lockdown, have you experienced any abuse as a result of an action from a family member?**						
No/Prefer not to say	221	65.6	60.2–70.6%	1.000	−	
Yes	116	34.4	29.4–39.8%	2.198	1.219–3.965	0.009
**During lockdown, have you been a witness to any abuse in your “bubble”?**						
No/Prefer not to say	264	78.3	73.6–82.6%	1.000	−	
Yes	73	21.7	17.4–26.4%	1.636	0.842–3.179	0.146

*Could not compute because of low number of responses. n, number of subjects; WHO-5, World Health Organization Well-Being Index 5.

## Discussion

### Key Findings and Comparison With Benchmark Data

COVID-19 pandemic and the lockdown restrictions have had a considerable impact on the psychological well-being of individuals. Our study is the first of its kind, conducted in Pakistan, assessing the well-being of individuals in Pakistan compared to other countries.

Based on the results depicting 76.1% of the population with moderate to high psychological distress, and 80.2% of the population with low mental well-being, it can be pronounced that almost three-quarters of the population in Pakistan under study was having difficulty adjusting to the new state of living during the lockdown due to the pandemic. This is almost two times higher when compared to the population of New Zealand with moderate to high psychosocial distress (30.3%) and severe anxiety (38.2%) (Every-Palmer et al., 2020).

The data available suggests significant diversity in the results based on gender. Most of the individuals with moderate to high psychological distress reported to be females (81.3%) compared to males (18.8%). Furthermore, the reported cases of moderate to severe anxiety (GAD > 10) were more prevalent in females (82.9%) than males (17.1%). This differs from the results seen for New Zealand reported by Every-Palmer et al. (2020) who reported that the gender gap was minimal. This difference is possibly due to the lower rates of transmission of COVID-19 in New Zealand (Summers et al., 2020), since like Pakistan, a cross sectional study conducted in Bangladesh where COVID-19 cases are higher, reported increased prevalence of psychosocial distress in females compared to males (Mamun and Griffiths, 2020). Similarly, a study from another country with a high number of COVID-19 cases, United Arab Emirates (UAE), also reported increased anxiety in females (51.7%) (Saddik et al., 2021). Our results also show significantly increased values for depression, anxiety and reduced well-being for females because women are generally more prone to diminished mental health as reported by several studies (Albert, 2015; Lim et al., 2018; Özdin and Özdin, 2020). Some plausible reasons for the increased mental health issues in females during the pandemic could be the rise in family quarrels, struggle of working women to execute their growing household responsibilities alongside their professions, having to care for family members, and having concerns about future while their partner is unemployed (Baig et al., 2020; Sigdel et al., 2020). Additionally, family violence (31.4%) being faced by women is also a contributing factor.

Women of Pakistan are facing domestic violence increasingly when compared to other countries; family violence in New Zealand was reported to be 4 times lesser (9%) (Every-Palmer et al., 2020) while that in Nigeria was also 3–4 times less (7.5–13.5%) (Ojeahere et al., 2021). The disparity in the cases of family violence reported in Pakistan and elsewhere is possibly due to the prevailing socio-cultural norms in Pakistan. According to a nationwide survey carried out by the Sustainable Social Development Organization of the government, from January 2020 to December 2020, domestic violence cases in Pakistan significantly increased during the lockdown; the cases of domestic and sexual abuse have doubled in the second half of 2020 when compared with the first half of the year (SSDO, 2020). There have been 1,422 cases of domestic violence, 9,401 cases of violence against females, and 4,321 cases of sexual abuse. There is a considerable gap between these statistics and the expected number of such cases because cases of workplace are not reported and are dealt within the company while a lot of cases are not reported at all. Hence, we must consider the limitation of this available data (The News International, 2021). Even before the pandemic, based on a study, two thirds of women in Pakistan reported domestic violence and consequently faced depression while staying silent due to their socio-cultural norms (Rabbani et al., 2008). With the implementation of lockdown, as women are forced to live in a confined space with their abuser, they not only face increased violence but also face difficulties contacting social, protective and health care services for help (Baig et al., 2020). The increased family violence reports could also be due to the fact that since mental health is stigmatized in Pakistan (Abdullah et al., 2021), less and less women ask for help (El-Nimr et al., 2021). This is an alarming situation which needs immediate attention because according to Rabbani et al. (2008) the psychosocial results of domestic violence are serious and include the use of drugs, alcohol consumption, depression, and suicidal attempts. It makes women feel vulnerable and diminishes their emotional stability and self-confidence.

Moreover, in the patriarchal society of Pakistan where 90% married women reportedly face physical or sexual abuse (Baig et al., 2020), domestic violence is considered to be a matter of the families rather than impeachment of human rights, which suggests there is a need for counseling on a societal level. Apart from the existing legislations for women’s protection based on Sharia law, government of Pakistan needs to adopt the efforts made in other countries. Like in India, Bangladesh and Iran, social service groups which work toward women empowerment, could be introduced. Additionally, health and family planning facilities could be developed to bridge the gap between the vulnerable women and the social service providers (Rabbani et al., 2008).

Some Arab countries, however, have reported even higher cases of family violence than Pakistan. A study involving most of the Arab countries reported that family violence increased from 39.6% before lockdown to 46.9% after lockdown (El-Nimr et al., 2021). This percentage of population is greater than that in Pakistan possibly because of the methodological limitations of our study and selection bias. Most of the women who face domestic violence in Pakistan are from lower socio-economic backgrounds (Rabbani et al., 2008) and hence possibly have lesser access to the internet while most of our population under study was from a better economic background with access to internet and computers or mobile phones.

Our study shows 21.4% people reporting suicide ideation which is higher than the 6% reported in New Zealand, 1.5% in Eswatini and 4.5% reported in Spain during the first lockdown (Every-Palmer et al., 2020; Mortier et al., 2021; Shongwe and Huang, 2021). Suicide cases are expected to rise further during lockdown as was seen in previous viral outbreaks (The Dawn, 2021). As mentioned earlier, from January 2020 to April 2020, during the early months of lockdown due to the pandemic, 29 suicide cases were reported in Pakistan (Mamun and Ullah, 2020). The plausible reasons for this surge could be that people of Pakistan and elsewhere have faced economic recession increasingly and according to studies there have been suicides possibly due to financial constraints, loss of employment during lockdown, media reporting of deaths due to COVID-19, exacerbation of pre-existing mental health issues, and in some cases the migrant’s inability to return home (John et al., 2020; Mamun and Ullah, 2020). Some people are even distressed due to their fear of contracting the infection while some are distressed due to the limited food supply during the lockdown (John et al., 2020; Mamun and Ullah, 2020). Additionally, there have been reports of teenagers and young adults attempting suicide due the results of videogames. The study reporting 3 cases of suicide due to a videogame has also reported that the excessive screen time during the lockdown while engaging in such activities has led to reduced mental health, increased suicidal ideation, and consequently increased suicidal attempts (Mamun et al., 2020). Notably, the difference in reported suicide data of our study and that from other countries could be due to a selection bias in our study which led to a higher percentage of suicidal ideation in the sample population compared to the general public.

Although majority of the effects of isolation were due to the lack of interaction, Osimo et al. (2021) reported that the cascade of negative psychological and behavioral effects triggered by the COVID-19 pandemic, were further regulated by personality traits, alexithymia, and resilience. Participants found to have higher levels of depression were amongst the high scorers during the evaluation for alexithymia. With reference to personality traits, higher emotional stability resulted in lower anxiety levels while increased openness to experiences resulted in a higher level of anxiety, indicating a causal relationship between alexithymia, personality traits, resilience, and depression due to confinement (Osimo et al., 2021).

These effects were also correlated with behavioral wellbeing, such as emotional eating, by Cecchetto et al. (2021). According to the results of their study, higher emotional eating was associated with higher BMI, alexithymia score, anxiety, and depression levels. Hence this indicates that the effects of isolation and lockdown include binge eating are directly modulated by alexithymia, resilience, and personality traits (Cecchetto et al., 2021).

### Assessment of Vulnerable Groups

People who are most vulnerable to the declining mental well-being include those who are dissatisfied with their living conditions (78.1%) (whether they live alone or with others), are unemployed, have underlying comorbidities, have a history of mental health issues, have experienced or witnessed domestic violence and people who have had suicidal thoughts.

Amongst people with moderate to high psychological distress, people living alone (1.9%) are most likely distressed due to the feeling of loneliness during quarantine (Seifert and Hassler, 2020) while people who are living along with others (98.1%) are possibly distressed due to the increase in disagreements and quarrels with living partners during the lockdown or increased violence within the family. This might be a result of loss of jobs during the pandemic which has caused additional stress with financial constraints and uncertainty during these unprecedented times (John et al., 2020; Mamun and Ullah, 2020). This makes people especially those with underlying mental health issues extremely vulnerable (Mamun and Ullah, 2020).

With the mental health care being shifted online due to the lockdown, people with past histories of mental health issues are receiving lesser attention and care. Based on a study, online appointments have reduced efficacy because there are mental issues which cannot be treated with online interaction and the health care professional might be unable to accord with the patient and due to the fact that online appointments are prone to technical issues (Feijt et al., 2020). Our study also supports this statement as it showed 13.3% individuals reporting previous mental illness related diagnosis while 40.5% were self-reported cases of deteriorating mental health. This reflects that there was a significant decline in the mental well-being of individuals during the pandemic.

Amongst people who reported comorbidities, 32.2% had moderate to high psychological distress, 31.5% had severe anxiety, and 29.7% had low mental well-being. This is in line with the study by Goodell et al. (2011) which states that medical comorbidities can lead to mental disorders since both have common risk factors including stress, childhood struggles, and socio-economic status. Regardless, when comparing those with low mental well-being in our study, people with underlying illnesses were less than those without, possibly because of people’s confidence in their doctor’s proficiency at treatment and diagnosis of COVID-19 as seen in a study conducted in Vietnam; it associated lower distress in individuals with increased confidence of patient in their healthcare system (Ngoc et al., 2020). The selection bias in our study also plays a significant role in the results here. Comorbidities are more likely to be prevalent in older individuals (Davis et al., 2011), and since most of our respondents were students aged between 18 and 29 years, less data was available to establish that the presence of comorbidities has no effect on psychosocial distress amongst people.

Students made up a significant portion (90.9%) of individuals among those with moderate or high K10 score. These students also reported comparatively higher scores on the GAD-7 index and lower WHO-5 scores compared to the rest of sample. These findings are consistent with the results seen in Australia; Lyons et al. (2020) reported that the mean K10 score among medical students in Australia during the COVID-19 pandemic was 20.6, which indicates moderate levels of psychological distress. It also highlighted the reasons for this remarkably high incidence of mental health issues among students; the most common concern being the impact COVID-19 pandemic has had on their studies followed by; the uncertainty about a return to normal life, family testing positive for COVID-19, being in self isolation and financial uncertainty (Lyons et al., 2020). Similar study was conducted on students in Bangladesh and, comparatively less stress and anxiety levels were reported, the reasons for the discrepancy between results of this study and ours could be that this study used a different scale [Depression, Anxiety and Stress Scale—21 Items (DASS-21)] (Khan et al., 2020). Other reasons include different sociodemographic status of students who participated; there were fewer female participants in the sample of this study (37%) compared to ours (79%) and as has already been discussed, females are at a higher risk of domestic violence and abuse which leads to higher psychological distress levels and anxiety (Khan et al., 2020). A study on medical students of a private university from February 2003, found out that prevalence of anxiety and depression was found among 60% of the students. Our study reports significantly higher prevalence of these disorders among students which proves the fact that COVID-19 pandemic has had a serious mental impact on students in Pakistan and it is imperative that concerned authorities cater to this issue (Inam et al., 2003).

### Comparison With International Studies

Lockdowns while being effective in curtailing the spread of COVID-19 are also putting the population at risk of deteriorating mental health. Even though Pakistan does not feature in the countries severely affected by the pandemic, the prevalence of mental distress and anxiety is comparable to international studies reporting negative implications of COVID-19 pandemic on mental well-being of general population (Banna et al., 2020; Every-Palmer et al., 2020; El-Nimr et al., 2021; Fornili et al., 2021). Females, students, individuals with underlying comorbidities and a history of mental health issues have shown to be more prone to the negative effects across several studies (Every-Palmer et al., 2020; Khan et al., 2020; Lyons et al., 2020; Özdin and Özdin, 2020; The News International, 2021). Increasing suicide ideation and incidence of domestic violence during the pandemic are also being reported by other studies from New Zealand, Spain, and Arab countries (Every-Palmer et al., 2020; Abdullah et al., 2021; The News International, 2021).

### Future Steps

Looking at these statistics, and the ongoing pandemic, it can be accepted that the lockdowns might continue. The decline in family and social contact, lesser entertainment options, job losses and financial uncertainty, and shifting of universities and schools to online platforms have all significantly contributed toward the aggravation in psychological distress, anxiety, family violence, suicidality, and well-being of individuals (Every-Palmer et al., 2020). Hence, there is a need for provision of psychosocial interventions and mass-media campaigns to increase awareness regarding the services available for people dealing with abuse or other mental health issues. Typing the words “Mental Health Pakistan” in PubMed query box gives 494 results in 2020 and 2021 alone, this rapid influx of data should be used by concerned organizations including the governments to counter this epidemic of mental health issues during these challenging times. Further research needs to be done, focused on individuals who are at a higher risk of deteriorating mental health (students, females, those with pre-existing mental health conditions, socioeconomically challenged families), governments and other international organizations should also play its role by providing incentives for research of this sorts. Newer research conducted must conceptualize the relationship between lockdown, personality traits and behavioral wellbeing to help improve COVID-related assessments.

Awareness campaigns on a national level are imperative to remove the stigma around mental health and these programs should help the public realize the severity of the issue and how it may lead to self-harm and suicide ideation. Sehat Sahulat Program has been launched, by some of the provincial governments in Pakistan, which aims at improving the quality of health care available to low-income households; however, among the treatment packages mental healthcare is not covered. Hence, adequate psychological support must be prioritized by the governments and should not be regarded as secondary compared to other health issues (Sehat Sahulat Program, 2021). Additionally, telehealth centers should be developed particularly in remote areas where people have access to quality e-therapies since most of the public in Pakistan either does not have access to the internet or does not know how to operate it.

### Limitations

Our study like any other study is not devoid of limitations. The study was based on an online survey, which creates a population bias, especially since in Pakistan, most people from remote areas and from lower socio demographics have restricted access to smartphones, laptops, or computers through which they could have filled the survey form (Nagra et al., 2021). Hence, our sample population is not representative of the general population. However, in a rapidly evolving pandemic situation, online survey was the most efficient method at hand.

Secondly, most of the respondents of our study are aged between 18 and 29 years and are students or recent graduates. Students and fresh graduates are known to have been highly affected during the pandemic due to closure of institutions and decline of economy due to less available jobs, resulting in a selection bias (Wang et al., 2020; Ali A. et al., 2021). Thirdly, most of the depression and anxiety cases were self-reported. Self-reporting is known to be less accurate for diagnosis of mental health as it generates higher point prevalence compared to a clinical evaluation for depression (Summers et al., 2020; Saddik et al., 2021), resulting in response bias. Lastly, this study is a cross-sectional study rather than a prospective study so it cannot be used to assess the causes of the onset, progress and results of anxiety and low mental well-being among the population of Pakistan.

## Conclusion

With the lockdown restrictions, psychosocial distress has become prevalent in Pakistan and elsewhere. The results from Pakistan are mostly along the lines with the results from other countries due to Pakistani government opting for multiple smart lockdowns to reduce pressure on its economy. Regardless, the isolation has had its negative implications which can only be curtailed by timely provision of psychosocial support, other support services to individuals facing abuse and suicide ideations, as well as basic supplies of personal protective equipment required by the healthcare workers.

## Data Availability Statement

The raw data supporting the conclusions of this article will be made available by the authors, without undue reservation.

## Ethics Statement

The studies involving human participants were reviewed and approved by Dow University Hospital-Ojha Campus. The patients/participants provided their written informed consent to participate in this study.

## Author Contributions

FY: conceptualization and funding acquisition. HJ, MAb, HN, and AK: data curation. MAs: formal analysis. FY, HJ, and SS: investigation. MAs and AK: methodology. FY, MAl, HJ, and AK: project administration. HJ, MAb, and HN: resources. MAs, SS, and HN: software. MAs, HN, and AK: validation. MAb and HN: visualization. HJ and SS: writing—original draft. FY, MAl, MAs, and AK: writing—review and editing. All authors contributed to the article and approved the submitted version.

## Conflict of Interest

The authors declare that the research was conducted in the absence of any commercial or financial relationships that could be construed as a potential conflict of interest.

## Publisher’s Note

All claims expressed in this article are solely those of the authors and do not necessarily represent those of their affiliated organizations, or those of the publisher, the editors and the reviewers. Any product that may be evaluated in this article, or claim that may be made by its manufacturer, is not guaranteed or endorsed by the publisher.
