# Supplementary material for: Psychological Distress, Anxiety, Family Violence, Suicidality, and Wellbeing in Pakistan During the COVID-19 Lockdown: A Cross-Sectional Study
Source: Front Psychol. 2022 Mar 15;13:830935. doi: 10.3389/fpsyg.2022.830935 (PMC8964640; doi:10.3389/fpsyg.2022.830935)
Supplement: Supplementary file 1 [file Table_1.DOCX]

| Supplementary Table 1: Psychosocial responses of the study participants (n=420). | | | |
| --- | --- | --- | --- |
| Variable | Frequency (n) | Percentages (%) | 95% confidence interval |
| **Living circumstances**  Family  Friends  Pet  Alone | 397  12  3  8 | 94.5  2.9  0.7  1.9 | 91.9–96.5%  1.5–4.9%  0.1–2.1%  0.8–3.7% |
| **Satisfaction levels with living bubble**  Dissatisfied  Neutral  Satisfied  Very satisfied | 114  221  52  33 | 27.1  52.6  12.4  7.9 | 22.9–31.7%  47.7–57.5%  9.4–15.9%  5.5–10.9% |
| **Level of contact with family/friends outside bubble**  Decreased  Stayed the same  Increased | 224  129  67 | 53.3  30.7  16.0 | 48.4–58.2%  26.3–35.4%  12.6–19.8% |
| **Overall, how things are currently progressing related to the pandemic?**  Getting better  Getting worse  Staying about the same | 76  194  150 | 18.1  46.2  35.7 | 14.5–22.1%  41.3–51.1%  31.1–40.5% |
| **How has it been to stay connected with family/friends outside bubble?**  Somewhat easy  Somewhat hard  Neither easy nor hard  Didn't try to stay connected  Extremely easy  Extremely hard | 97  75  128  42  46  32 | 23.1  17.9  30.5  10.0  11.0  7.6 | 19.1–27.4%  14.3–21.9%  26.1–35.1%  7.3–13.3%  8.1–14.3%  5.3–10.6% |
| **How are you and the people you are living with getting along with each other?**  Badly  Very badly  Neither badly nor well  Well  Very well | 35  10  129  177  69 | 8.3  2.4  30.7  42.1  16.4 | 5.9–11.4%  1.1–4.3%  26.3–35.4%  37.4–47.0%  13.0–20.3% |
| **How often have you felt lonely or isolated?**  Never  Rarely  Sometimes  Often  Always | 37  63  137  121  62 | 8.8  15.0  32.6  28.8  14.8 | 6.3–11.9%  11.7–18.8%  28.2–37.3%  24.5–33.4%  11.5–18.5% |
| **How often have you been able to enjoy yourself during the social distancing period?**  Never  Sometimes  Often  Very often  Always | 34  212  88  48  38 | 8.1  50.5  21.0  11.4  9.0 | 5.7–11.1%  45.6–55.4%  17.2–25.2%  8.5–14.9%  6.5–12.2% |
| **Do you have a job now?**  Yes  No  Self employed  Own business | 29  382  5  4 | 6.9  90.9  1.2  1.0 | 4.7–9.8%  87.8–93.5%  0.4–2.8%  0.3–2.4% |
| **Lost job during the lockdown**  Yes  No  Never had a job | 12  42  366 | 2.9  10.0  87.1 | 1.5–4.9%  7.3–13.3%  83.6–90.2% |
| **Do you have a medical condition that makes you more vulnerable to COVID-19 infection?**  No  Yes  Prefer not to say | 351  45  24 | 83.6  10.7  5.7 | 79.7–87.0%  7.9–14.1%  3.7–8.4% |
| **Do you live with someone who has a medical condition that makes them more vulnerable to COVID-19 infection?**  No  Yes  Prefer not to say | 273  123  24 | 65.0  29.3  5.7 | 60.2–69.6%  25.0–33.9%  3.7–8.4% |
| **Smoking status**  I have never been a smoker  I am an ex-smoker  I smoke cigarettes less than once a week  I smoke cigarettes at least once a week, but not daily  I smoke cigarettes every day | 397  7  5  7  4 | 94.5  1.7  1.2  1.7  1.0 | 91.9–96.5%  0.7–3.4%  0.4–2.8%  0.7–3.4%  0.3–2.4% |
| **History of Depression (self-reported)**  Yes  No | 49  371 | 11.7  88.3 | 8.8–15.1%  84.9–91.2% |
| **History of any mental illness diagnosed by a doctor or psychologist?**  No  Yes  Prefer not to say | 335  56  29 | 79.8  13.3  6.9 | 75.6–83.5%  10.2–17.0%  4.7–9.8% |
| **During lockdown, how is your mental health compared to usual?**  Better than usual  Much better than usual  Much worse than usual  Prefer not to say  Same as usual  Worse than usual | 45  11  39  18  137  170 | 10.7  2.6  9.3  4.3  32.6  40.5 | 7.9–14.1%  1.3–4.6%  6.7–12.5%  2.6–6.7%  28.2–37.3%  35.7–45.3% |
| **During the lockdown, have you seriously thought about ending your own life?**  No  Yes  Prefer not to say | 310  90  20 | 73.8  21.4  4.8 | 69.3–78.0%  17.6–25.7%  2.9–7.3% |
| **During the lockdown, have you made plans to end your own life?**  No  Yes  Prefer not to say | 344  62  14 | 81.9  14.8  3.3 | 77.9–85.5%  11.5–18.5%  1.8–5.5% |
| **During the lockdown, have you made an attempt to end your own life?**  No  Yes  Prefer not to say | 388  18  14 | 92.4  4.3  3.3 | 89.4–94.7%  2.6–6.7%  1.8–5.5% |
| **During lockdown, have you experienced any abuse as a result of an action from a family member?**  No  Yes  Prefer not to say | 278  132  10 | 66.2  31.4  2.4 | 61.4–70.7%  27.0–36.1%  1.1–4.3% |
| **Family harm during lockdown***  Physical assault  Insulting, harassed and threatening behavior  Sexual assault  Frightened by a family member | 44  46  6  36 | 34.8  33.3  4.5  27.3 | 26.8–43.6%  25.4–42.1%  1.7–9.6%  19.9–35.7% |
| **During lockdown, have you been a witness to any abuse in your 'bubble'?**  No  Yes  Prefer not to say | 315  85  20 | 75.0  20.2  4.8 | 70.6–79.1%  16.5–24.4%  2.9–7.3% |
| **Have you experienced any 'silver linings' or positive aspects during the COVID-19 lockdown?**  No  Yes, for me personally  Yes, for the society | 166  177  77 | 39.5  42.1  18.3 | 34.8–44.4%  37.4–47.0%  14.7–22.4% |
| **K10**  >12 (moderate or high)  <12 (low or mild) | 100  320 | 23.8  76.2 | 19.8–28.2%  71.8–80.2% |
| **GAD-7**  >10 (high anxiety score)  <10 (low anxiety score) | 204  216 | 48.6  51.4 | 43.7–53.5%  46.5–56.3% |
| **WHO-5**  <13 (low well-being score)  >13 (high well-being score) | 337  83 | 19.8  80.2 | 16.1–23.9%  76.1–83.9% |
| * Indicates (n=132) assault/abuse responses only.  n: number of subjects. K10: Kessler Psychological Distress Scale; GAD-7: Generalized Anxiety Disorder Assessment; WHO-5: World Health Organization Well-Being Index 5. | | | |
